# Feeding Calves with Pasteurized Colostrum and Milk Has a Positive Long-Term Effect on Their Productive Performance

**DOI:** 10.3390/ani10091494

**Published:** 2020-08-24

**Authors:** Ramon Armengol, Lorenzo Fraile

**Affiliations:** 1 Department of Animal Science, ETSEA, University of Lleida, 25198 Lleida, Spain; rarmengol@ca.udl.cat; 2 Agrotecnio Research Center, ETSEA, University of Lleida, 25198 Lleida, Spain

**Keywords:** colostrum, milk, pasteurize, long-term effects, dairy

## Abstract

**Simple Summary:**

The main objective of this study was to observe whether feeding female calves with pasteurized colostrum and cow’s milk improved future reproductive performance, productive parameters, and health over the course of the heifer-rearing process and the three first lactations. During the heifer-rearing period, growth and health parameters were recorded in two populations: one that received pasteurized colostrum and milk during the first 21 days of life (Experimental Group-P) and one that received unpasteurized colostrum and milk (Control Group-NP). During the cows’ life, productive (305-d milk yield), reproductive (artificial insemination per pregnancy and calving interval), and health parameters (milk somatic cell count), as well as age at culling, were recorded. Feeding on-farm pasteurized colostrum and milk during the first 21 days of life reduced morbidity of bovine respiratory disease during the first year of life and diarrhea during the first 180 days of life. Moreover, it increased body weight at calving during the first three lactations. It also significantly increased milk production during the first lactation. Thus, feeding female dairy calves with pasteurized colostrum and milk improved health and productive parameters in heifers and cows, and has a positive long-term effect on cow’s production parameters. This practice is recommended as a general approach to improving performance in dairy herds.

**Abstract:**

Female calves, checked for serum total protein ≥ 5.8 g/dL before 5 days of life, entered the study at 22 days of age after having received pasteurized colostrum and milk (P group, *n* = 127), or non-pasteurized colostrum and milk (NP group, *n* = 134). During the heifer-rearing period, productive (body weight; BW) and health parameters (bovine respiratory disease (BRD) and diarrhea) were recorded. Productive (305-d milk yield), reproductive (AI per pregnancy and calving interval), and health parameters (milk somatic cell count; SCC/mL), as well as age at culling, were recorded in a follow-up study. Feeding on-farm pasteurized colostrum and milk during the first 21 days of life reduced morbidity of bovine respiratory disease during the first year of life and diarrhea during the first 180 days of life. Moreover, it increased BW at calving during the first three lactations. It also significantly increases milk production during the first lactation. However, there were no differences in relation to reproductive performance and health of cows in the NP or P group. These results highlight that feeding calves with pasteurized colostrum and milk could improve health and production parameters throughout the heifer-rearing process and during their first lactation.

## 1. Introduction

The ingestion of colostrum by newborn calves, during the first hours of life, is critical for its survival and development during the neonatal period [1,2]. An appropriate ingestion is guaranteed if high-quality, hygienic colostrum (>50 g IgG/L, <100,000 cfu/mL of total bacterial counts and <10,000 cfu/mL of fecal coliforms counts) is administered in sufficient quantities. If these requirements for colostrum ingestion are met, calves should achieve levels of serum IgGs over 10 g/L during the first 24–48 h of life [2,3]. It is well known that inadequate serum levels of IgG (IgG concentration < 10g/L) lead to failure of passive transfer (FPT), which is a risk factor for greater incidences of pathologies and mortality during the first weeks of life [4]. For this reason, management, administration, and storage of colostrum have been studied for years to determine the conditions necessary for passive transfer (PT) [5,6].

The calves’ feeding systems and diet can be highly variable during their pre-weaning phase [7]. Thus, any feeding system based on either milk or milk replacers is usually enriched with concentrated feed, a source of fiber (straw or hay) and water ad libitum. An ideal calf feeding system should allow growth and maturation while reducing morbidity and mortality.

Colostrum and cow’s milk, or their substitutes, can be a direct or indirect source of disease, either due to excess bacterial load or through pathogen transmission [8,9]. Moreover, high bacterial loads reduce the amount of total protein absorbed after colostrum ingestion [10]. Therefore, colostrum, milk, or MR, must have a guaranteed level of hygiene without a negative impact on their quality. A common method of long-term preservation of colostrum at the farm level is freezing, because it does not negatively affect its quality (as long as it thaws at <60 °C) and minimizes bacterial growth [1,2]. Measuring the quality of the colostrum of cows and freeze the excess of good quality colostrum may help to guarantee that all newborn calves will receive an appropriate passive transfer of immunity, even in those cases where cows have produced little quantity and/or quality of colostrum. Pasteurization has been used to preserve milk quality for human consumption since the beginning of the 1900s [11]. More recently, on-farm pasteurization of colostrum and milk in calf feeding has become popular [12,13]. The pasteurization techniques for colostrum and cow’s milk differ from those used for human consumption, since exceedingly high temperatures can denature proteins, such as immunoglobulins in the case of colostrum, affecting its potential to fulfil its main objective, namely passive transfer immunity [5]. Moreover, colostrum and milk pasteurization has been demonstrated to reduce bacterial contamination [5,14,15], increase IgG transfer [5,16], improve health status and decrease mortality during the first weeks of a calf’s life [17].

To the authors’ knowledge, there are no studies on the effect of pasteurized colostrum or milk on future productive and reproductive parameters over the first lactation in cows. The main objective of this study was to observe whether the feeding of calves with pasteurized colostrum and cow’s milk affects their future reproductive performance, productive parameters, and health over the first three lactations compared to calves fed with non-pasteurized colostrum and cow’s milk under the same environmental and management conditions.

## 2. Materials and Methods

### 2.1. Ethical Statement

All experimental procedures were approved by the Ethics Committee for Animal Experimentation of the University of Lleida and performed in accordance with authorization 10343 issued by the Catalan Department of Agriculture, Livestock, Fisheries, and Food (Section of biodiversity and hunting).

### 2.2. Animals and Farm

The study was carried out at a dairy housing 330 lactating Holstein cows with an average production of 11,100 kg of milk (3.6% fat and 3.3% protein) per cow. This farm was located in Lleida (Northeast Spain). Cows were milked three times daily (at 04:00, 12:00, and 20:00 h). Milk from each cow was sampled and analyzed for milk quality (fat, protein, and lactose concentration) and somatic cell count (SCC) by technicians from the Central Laboratory for Milk Recording (ALLIC, Catalonia, Cabrils, Spain) once a month. Cows were moved three weeks before calving to a facility where parturition in groups took place (5–10 cows). The first milking of colostrum was carried out between 30–90 min after calving.

Newborn calves were removed from the pen immediately after calving to avoid suckling. The first phase of the study [17] was completed at 21 days of life. In this follow-up study, only female Holsteins entered the second phase of the study at 22 days of life, when the first grouping occurred. These females were already randomly assigned to two groups at first colostrum intake (≤3 h after birth): NP (non-pasteurized colostrum and milk) and P (pasteurized colostrum and milk). Colostrum collected at the first milking was tested for quality (specific gravity ≥ 1.065) as described in a previous paper [17]. Female calves assigned to the NP Group (*n* = 148) were fed frozen (−20 °C) colostrum that was previously reheated to 40 °C (6–8 L during the first 12 h of life). They were then fed raw milk from the bulk tank that was also reheated to 40 °C (1.3–2.3 L every 12 h). Calves assigned to the P Group (*n* = 141 females) were under the same feeding protocol, but colostrum and milk collected from the bulk tank had been pasteurized. For the P group, detailed information about colostrum and milk pasteurization was previously reported [17].

**Figure 1:**
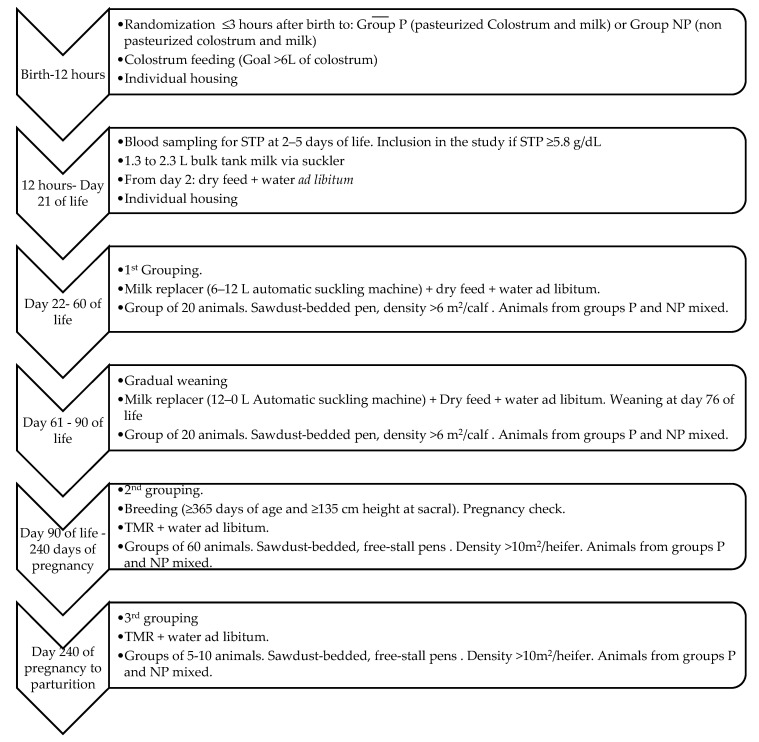
Management, nutritional, and husbandry protocols used throughout the calf- and heifer-rearing process of the study. All calves in Group P (pasteurized colostrum and milk) and Group NP (non-pasteurized colostrum and milk) were mixed from day 22 of life onwards. STP, serum total protein; TMR, total mixed ration.

Blood samples were drawn from all calves at 2–5 days of life, and serum was obtained through centrifugation. Serum total protein (STP) (g/dL) was determined using a commercially available refractometer (AtagoMastersur/NE, Atago U.S.A. Inc., Bellevue, WA, USA). Only female Holstein calves, with an STP value ≥ 5.8 g/dL, associated with appropriate colostrum ingestion [4,18,19], entered the study. All calves were offered fresh water and dry feed ad libitum from day 2 until the end of the study. Calves were individually housed during the first 21 days of life. From day 22 until weaning at day 76, calves were moved into groups of 20 animals in a sawdust-bedded pen. The group density was 6 m^2^/calf during the pre-weaning period. This grouping was assigned randomly, mixing animals from groups NP and P to avoid any confounding effects due to housing. Feeding throughout this period was based on MR supplied by an automatic suckling machine (The Dairy Feed J V640+, GEA, Düsseldorf, Germany). MR daily intake was increased from day 22 (6 L/day) until day 60 with a maximum of 12 L/day. This feeding system allows each calf a maximum volume of 3 L per intake and a minimum time interval between intakes of two hours. Finally, daily MR intake decreased from day 61 until weaning at day 76. The heifers were comingled in groups of 60 animals per pen between 90 to 240 days of pregnancy, maintaining the initial groups together. Housing was in sawdust-bedded, free-stall pens and a minimum density of >10 m^2^/heifer was always guaranteed. Feeding after weaning included a total mixed ration based on hay, rye grass silage, alfalfa, corn pastone, and water ad libitum. The timeline of the calf- and heifer-rearing process is detailed in Figure 1.

### 2.3. Health Monitoring of Heifers and Cows

All females underwent a clinical observation and milk consumption monitoring (The Dairy Feed J V640+ software, GEA, Germany) every 24 h until 76 days of life (weaning age). Furthermore, a clinical score for BRD was applied twice weekly [20]. After weaning, heifers were under clinical observation every 48 h until 365 days of life. Rectal temperature measurements, auscultation, and thoracic ultrasound were carried out when the veterinarian suspected sickness. In summary, bovine respiratory disease (BRD) and diarrhea were monitored during the first year of life, although diarrhea was only observed during the first 180 days of life.

Health monitoring of cows was carried out daily using a trigger alarm from farm-specific software (Afifarm, Afimilk, Israel National Trail Kibbutz Afikim, 1514800, Israel). Thus, cows were clinically checked if their milk production and their activity was lower than expected and/or milk conductivity was greater than expected, using baseline levels set in the software as a reference. Clinical checkup of cows included auscultation and examination of the respiratory, digestive, and reproductive tracts, and legs, udder, rectal temperature, and blood concentration of beta-hydroxybutyrate. Mastitis incidence was considered as a key parameter to control for cow’s health since it can occur at any time during the lifetime of a cow and its prevalence is high in dairy herds. For this study, we decided to consider individual monthly milk SCC as a health parameter since it includes clinical but also subclinical mastitis [21]. SCC average value for each parturition was calculated as the arithmetic mean of monthly values over each lactation.

### 2.4. Reproductive Monitoring of Heifers and Cows

Heifers were placed in the breeding pen at the age of 365 days and at a minimum sacral height of 135 cm. After a minimum of 10 days in the breeding pen, heifers were artificially inseminated (AI) when heat was detected either visually or using a tail marking with chalk strategy. Breeding programs, including hormonal synchronization, were not routinely used in heifers. If heifers were not inseminated by the age of 425 days, transrectal ultrasonography (Easi Scan, 4.5–8.5 MHz; BCF Technology Ltd., Scotland) was used to detect ovary structures and then apply hormonal therapy if needed. Briefly, in the presence of a corpus luteum ≥ 2 cm and a follicle ≥ 1 cm, a dose of prostaglandin F2α was applied, and heifers were inseminated at detected oestrus. If there was no corpus luteum or the size was <2 cm, the therapy was based on an ovsynch protocol with a progesterone device. The average number of AI per pregnancy was recorded to detect differences between the NP and P groups. The number of AI per pregnancy is defined as the average total number of AI over the total number of pregnant heifers or cows of the group during a period.

Cows’ reproduction strategy was based on routine hormonal protocols, double ovsynch and ovsynch as a resynch [22], and a computerized pedometry system (Afimilk, Israel National Trail, Kibbutz Afikim, 1514800, Israel). A voluntary waiting period (VWP) of 85 days was established for all the cows. Transrectal ultrasonography was used to perform pregnancy diagnosis at 28 to 35 days post-AI in both heifers and cows, to detect uterine and/or ovarian disorders such as clinical endometritis, pyometra, ovarian cysts, and ovarian cyclicity failure. This reproductive control was carried out every Tuesday and Friday and also included postpartum examination (1–21 days postpartum) and diagnosis and treatment of non-cycling or silent oestrus cows. Two confirmations of pregnancy diagnosis were carried out at day 90 and 200 of pregnancy. Breeding management was carried out by AI with Holstein semen and performed by a highly trained technician or veterinarians specialized in cattle reproduction (Lleidavet S.L.P, Alpicat, Lleida, Spain). Reproductive performance in cows was assessed through the average number of AI per pregnancy and calving interval [23,24]. Calving interval is defined as the average number of days elapsed between current and previous calving.

### 2.5. Monitoring of Body Weight

Calves were weighed immediately at birth prior to colostrum ingestion using a set of scales (WA08, Meyer-Brakenberg Gmbh & Co., Extertal, Germany). Scale accuracy was ±0.1 kg. We were not able to weigh the calves and heifers during the rearing process until they had their first parturition due to reasons related to farm management and labor. Cows’ BW was measured three times a day (Afimilk, Israel National Trail, Kibbutz Afikim, 1514800, Israel). The scale was located under the base of the sorting door box, so that cows could be weighed every time after milking. The cows’ BW data in this study is after parturition and is the average from the first seven days in milk (DIM) (*n* = 21).

### 2.6. Fate and Cause of Culling for Heifers and Cows

The fate of culled cows was classified into two groups: slaughtered or dead on the farm. There was no sale of animals for further productive purposes. The cause of culling was established based on the classification proposed by the USDA in the National Animal Health Monitoring Scheme and recent papers [25,26,27]. The cause of culling was established by consensus between the veterinarian and the farmer, taking into account the veterinary diagnosis and the productive, genetic improvement and milk quality criteria of the farmer. Although the farm software was able to capture different possible causes of culling, the database only retains the primary cause of culling in the animal’s individual history. The causes of culling were reproductive, mammary gland, production, locomotor, metabolic/digestive, respiratory, dystocia/obstetrics, accidental, infectious, and unknown [27].

### 2.7. Collection of Data

Clinical, reproduction, production, and management data were recorded using specific software (Afifarm, Afimilk, Israel National Trail, Kibbutz Afikim, 1514800, Israel). The following individual data for the heifers and cows were recorded from the herd database: Identification of the heifer-cow, date of birth, date of artificial insemination, pregnancy diagnosis, disease diagnosed, monthly SCC/mL, date of calving, date of death, fate (slaughtered or dead at the farm), cause of culling, age at death and number of lactations at culling. Moreover, individual production data for each cow under study included BW at birth (kg), BW after parturition (kg) and 305-d milk yield (kg milk produced during a standardized lactation).

### 2.8. Statistical Analyses

All statistical analyses were carried out using SAS V.9.1.3 (SAS Institute Inc., Cary, NC, USA). For all analyses, the individual calf was used as the experimental unit. The significance level was set at 0.05, with statistical tendencies reported when the *p*-value < 0.10. The explanatory variable was the pasteurization process (NP and P). The outcome variables were its effect on BW, reproductive performance (age at first parturition, calving-calving interval and the number of inseminations needed to reach pregnancy), 305-d milk yield (kg), milk quality (SCC/mL), the morbidity of respiratory and diarrhea during the first year of life and culling (fate and cause). Morbidity was calculated as the number of cases of respiratory illness or diarrhea observed divided by the number of calves during a period in the NP and P group, respectively. In the cases of heifers, these parameters were studied, splitting the population into heifers that reached first parturition and those that did not. The categorical variables included in the statistical analyses were: Pasteurization status (NP/P), BRD or diarrhea (yes/no), culling (yes/no), the fate of culling (slaughter/dead in the farm), and cause of culling (BRD, diarrhea and other). The continuous variables used in the statistical analysis were: BW (kg), age at first parturition (d), milk quality (SCC/mL), 305-d milk yield (kg), calving-calving interval (d) and the number of inseminations needed to achieve pregnancy. Shapiro Wilk’s and Levene tests were used to evaluate the normality of the distribution of the continuous variables and the homogeneity of variances, respectively. Descriptive statistics were calculated, focusing on the number of parturitions and pasteurization status (Table 1).

Statistical analyses were performed to test the association between the two experimental groups (NP and P) by the number of parturitions with the outcome variables (Table 1). The association between nominal variables with continuous non-normally distributed variables were analyzed using the Wilcoxon test (with the Mann–Whitney U test to compare each pair of values). To analyze the association between continuous normally distributed variables and nominal variables, an ANOVA test (with Student’s *t*-test to compare each pair of values) was used. Finally, contingency tables (chi-square or Fischer’s exact tests) were used to assess the association between categorical variables.

A sample size calculation was carried out to achieve sufficient statistical power to detect differences between experimental groups based on biologically relevant differences in production parameters (BW at first parturition and 305-d milk yield). Thus, 99 calves per group were sufficient sample size to detect 20 kg of difference in BW at first parturition (from 490 ± 50 to 510 ± 50 kg) and 400 kg (from 11,000 ± 1000 to 11,400 ± 1000 kg) in 305-d milk yield, respectively between groups with a confidence level of 95%.

## 3. Results

A total of 261 female Holstein calves ultimately entered the study (134 in the NP group and 127 in the P group). Fourteen animals were excluded from each group (28 animals in total), since their STP value was <5.8 g/dL. The percentage of calves with FPT was 9.5 and 9.9 in the NP and P group, respectively. These percentages agree with the expected value for this farm (10.0% for the last three years). Of these, 216 reached at least first parturition (NP = 110 and P = 106), 184 reached second parturition (NP = 90 and P = 94) and 139 reached third parturition (NP = 71 and P = 68). We did not observe significant differences in the percentage of animals reaching the first, second, or third parturition between the two groups. Raw data of this study are available in Supplementary S1 for interested readers.

### 3.1. Reproductive Performance

The average number of inseminations needed to achieve the first pregnancy was 1.54 and 1.55 for the NP and P groups (*p* = 0.91), respectively. We did not observe significant differences when comparing age at first parturition between the NP and P groups (*p* = 0.99). Moreover, no significant differences in the number of AI needed to achieve second (*p* = 0.73) and third pregnancy (*p* = 0.63) were observed. Finally, we did not observe significant differences in calving intervals for the two periods studied, either the first-to-second or the second-to-third lactation (Table 1).

### 3.2. Productive Performance

**Figure 2:**
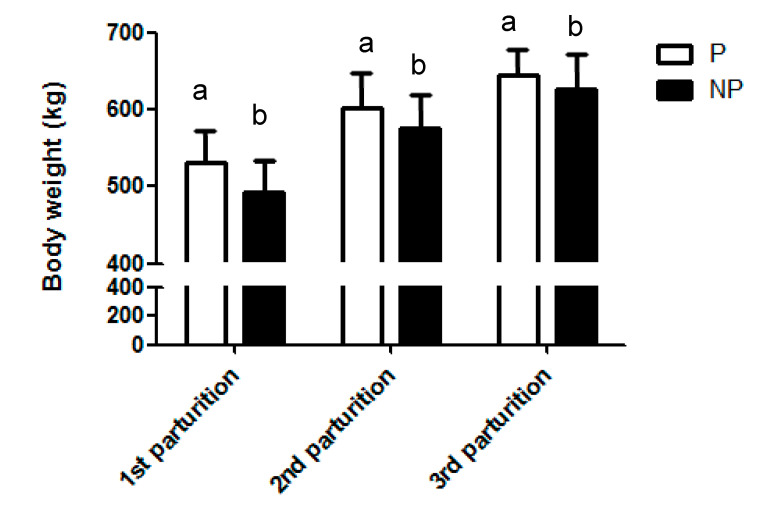
Evolution of BW (kg) depending on the number of parturition and experimental Group (NP vs. P Group). Bars with different superscripts (a, b) showed statistically significant differences (*p* < 0.05).

Body weight (BW) at birth was very similar (*p* = 0.98) between the NP (38.7 ± 1.1 kg) and P groups (38.6 ± 1.1 kg). Animals in the NP group were significantly lighter at first calving (492.6 ± 3.8 kg) compared to P group (531.7 ± 3.8 kg) and this difference was also present in the subsequent calvings (575.1 ± 4.6 vs. 602.0 ± 4.6 kg at second calving and 626.4 ± 5.3 vs. 645.9 ± 5.3 kg at third calving) (*p* <0.01) (Table 1 and Figure 2).

**Figure 3:**
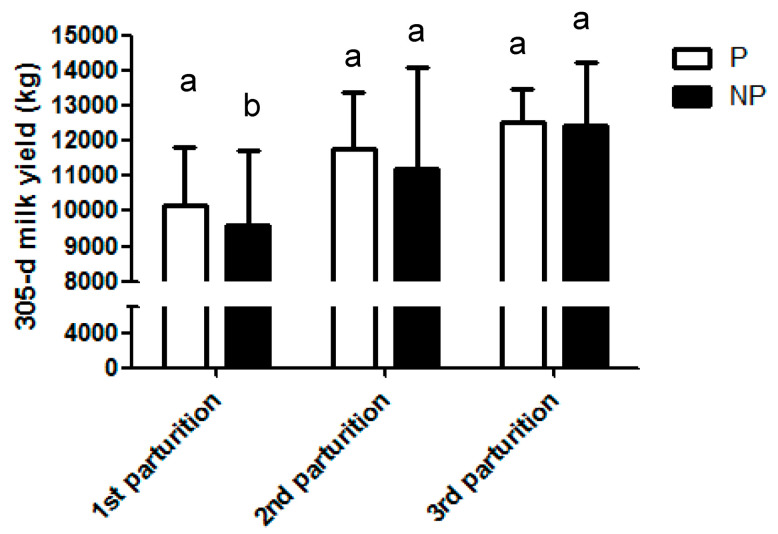
305-day milk yield (kg) depending on the number of parturition and experimental Group (NP vs. P Group). Bars with different superscripts (a, b) showed statistically significant differences (*p* <0.05).

The 305-d milk yield was analyzed during the first, second, and third lactation. We observed that the NP Group significantly produced 573 kg less milk (9574 ± 209 kg) compared to cows belonging to the P Group (10,147 ± 161 kg) during the first lactation (*p* = 0.04). Cows from the P Group continued producing more milk during the second lactation (11,754 ± 168 kg) compared to NP cows (11,193 ± 314 kg), but without observing significant differences between groups (*p* = 0.66). Both groups (NP and P) also produced a similar amount of milk (*p* = 0.68) in the third lactation (Table 1 and Figure 3).

### 3.3. Health Performance

Health during heifers’ rearing process was monitored considering BRD morbidity during the first year of life and diarrhea during the first 180 days of life. In heifers that never reached first parturition (NP group = 24 vs. P group = 21), morbidity from both diarrhea (*p* = 0.04) and BRD (*p* = 0.28) was lower in the P than in the NP group (4.8% vs. 26.1% for diarrhea and 38.1% vs. 56.5% in BRD), although in the case of BRD the differences were not significant. Similar trends were seen in animals that reached first parturition and differences were significant for both diseases (BRD, 10.4% vs. 21.8%; *p* = 0.02 and diarrhea, 4.7% vs. 12.7%; *p* = 0.03 (Table 1). If data from heifers reaching parturition and those not reaching the first parturition are merged, repetition of diarrhea episodes was not observed during the study, but heifers could suffer more than one episode or BRD. The number of episodes/animal of sickness in calves and heifers was never different for the P and NP group for BRD (*p* = 0.20) and diarrhea (*p* = 1.00).

Udder health was studied regarding in terms of the average SCC during first, second and third lactation. No significant differences were observed between NP and P groups. Complete health data are detailed in Table 1.

### 3.4. Culling

Heifers that did not reach first parturition from the NP group tended to be culled earlier than those in the P group (*p* = 0.07). Percentage of heifers dead on the farm tended to be greater in the NP group compared to the P group (*p* = 0.08) (Table 1). There were no significant differences between NP and P groups when analyzing reasons for culling. Heifers that reached at least first parturition were culled at an average age of 1265.2 days in the NP group and 1234.1 in the P group, showing no significant differences (*p* = 0.62). There were no significant differences between NP and P groups in the fate of culled cows (slaughter vs. dead in the farm) and cause of culling.

## 4. Discussion

Pasteurization of colostrum with a specific gravity ≥ 1065 and milk is an effective method for reducing pathogen exposure in calves, as well as morbidity and mortality during the first 21 days of life [17]. It is well known that appropriate colostrum intake during the first hours of a calf’s life is crucial to prevent diseases, reduce mortality, and maximize growth during the pre-weaning period [2,17]. The present study is considered a follow-up to our previous research [17] with the main goal of describing the effect of feeding pasteurized colostrum and cow milk during the first 21 days of life on reproductive performance, productive parameters and health over the course of the heifer-rearing process and the three first lactations. This type of follow-up study is extremely complicated to carry out under field conditions because the same animals must be monitored for a long period (6 years). We were able to carry out this study on a single farm. It would have been desirable to include more farms, but it would have been extremely difficult to recruit additional farms for this study due to the long period of monitoring. The fact that only a single farm was included is a limitation of the study, but the consistency of the record-keeping on this farm is outstanding. Moreover, management and composition of early feeding or housing in the early stages of heifers’ life were identical for both groups avoiding confusion factors because these aspects have been demonstrated to have an impact on future growth, health, reproduction, and milk yield [28,29]. On the other hand, our study only included animals that have received adequate amounts of colostrum (STP value ≥ 5.8 g/dL). Thus, our research work is focused on the improvement in productive, reproductive and health parameters that can be achieved in cows by applying pasteurization on farms where the ingestion of good quality colostrum is guaranteed in most of the animals. In those farms with a high percentage of FPT, the first goal must be to achieve the appropriate colostrum ingestion in calves.

### 4.1. Reproductive Performance

Reproductive performance in heifers was studied at first parturition. We did not observe significant differences between the two groups in terms of the number of AI per pregnancy, probably because Holstein heifers, raised on this type of farm, meet all reference standards with respect to a mature cow’s BW, 55–60% at 1st AI and 85% after first parturition [30]. Reproductive performance in cows was not different between the P and NP groups of animals, either during their heifer period or after the second parturition. The number of AI per pregnancy can be influenced by many farm factors such as the technician, bull, environment [31,32], heat detection [32] or disease [33,34]. In this study, all of these factors were the same for both groups. This indicates that, at least when the conditions of feeding, handling, and accommodation for replacement heifers and cows are guaranteed, feeding with pasteurized colostrum and cow’s milk is not a significant factor in achieving better reproductive results.

### 4.2. Productive Performance

The main objective during the rearing process of a heifer must be to achieve adequate body size and weight at a certain age, but also to guarantee a certain status of maturity at both first AI and at first parturition to maximize efficiency in the process and future milk production [35]. It has been described that an increase in average daily gain during the pre-weaning period is associated with an increase in milk yield during the first lactation [36,37]. During this study, we could only weigh calves at birth and cows immediately after parturition. Unfortunately, the farmers did not allow us to weigh calves and heifers during the rearing process due to the extra labor costs. The results indicate that heifers fed pasteurized colostrum and milk showed significantly greater BW than those calves fed non-pasteurized colostrum and milk during three lactation periods. It must be highlighted that cows are not only producing milk and becoming pregnant, but also growing, at least until the beginning of the third lactation, when they can be considered mature cows [38]. Mature BW was set at 650 kg on this farm. Heifers from the NP and P group reached an average mature BW after parturition of 75.7% and 81.8%, respectively, and this may be one of the reasons why heifers from the P group significantly produced more milk during the first lactation. In summary, as previously reported, heifers with better growth produced more milk [37,39]. In a previous study [40] about long-term impacts of feeding mixed heat-treated colostrum, there was no difference in milk production during the first or second lactation. However, a direct comparison to our study is not possible because calves also received pasteurized whole cows’ milk during the first 21 days of life and the colostrum used was from the same dam and was never pooled. These differences make comparisons unfeasible. Whatever the case, we believe that research should be carried out in the future to confirm our results. 

### 4.3. Health Performance

BRD during the first year of life and diarrhea during the first 180 days of life are the two most common pathologies diagnosed during the heifer rearing period [4,41]. They may cause not only mortality, but a negative impact on growth and future milk production as well [37,42,43]. We studied two segments of the population during the rearing process: heifers that reached the first parturition and heifers that did not reach first parturition. The objective of studying these two populations separately was to determine if there were differences in morbidity. It is clear that heifers that never reached the first parturition suffered more BRD and/or diarrhea than those that became cows, showing that there are direct or indirect causes of culling during the rearing process as previously reported [42,43]. Furthermore, our results always showed lower morbidity for the animals fed pasteurized colostrum and milk compared to those fed non-pasteurized products in both populations. The number of cases of BRD per animal during the rearing period was low and not different between groups. We believe that strict control of the treatment and recovery of sick animals in this study may explain this result. Finally, female calves included in this study, from day 22 of life onwards, were grouped by mixing animals from groups P and NP due to reasons of farm management. We believe that commingling calves from P and NP may have compensated for some of the beneficial effects that the heat-treated colostrum and milk had during the first 21 days of life. Another potential explanation could be that feeding calves with pasteurized colostrum and milk during the first 21 days of life does not affect the number of cases of BRD per animal after this period.

We chose to monitor health when heifers became cows using SCC for three reasons. Mastitis is a multifactorial disease that is highly dependent on the immune system [44]. Second, it is the most frequent disease in dairy cows [45], and it fully covers the day-to-day health status of the cow because it includes clinical as well as subclinical and chronic problems [21,46,47]. Other diseases such as uterine or metabolic diseases were not included because it was not possible to diagnose subclinical forms for these on the farm. We did not observe any significant differences in SCC between P and NP groups during any of the three lactation periods under study. While it has been reported that milk fat and protein can be affected by disease morbidity in the first four months of age [42], we found no information regarding SCC.

### 4.4. Culling

When we analyzed the culling data according to fate (slaughter or dead on the farm), days of life at culling and life milk yield, no significant differences were observed between NP and P groups. In a previous study, it was observed that the use of mixed heat-treated colostrum had no effect on removal from a herd [40]. It has also been reported that diarrhea is not associated with the likelihood of finishing the first lactation [48], but the same author reported that heifers that suffered four or more BRD episodes had a greater risk of not completing the first lactation. It is important to note that in our study, the average number of BRD across all the calves and heifers included in the study was always below 1.5 episodes. Waltner-Toews et al. [49] reported a negative impact in short- (mortality before 90 days of life) and long-term (age at first calving) parameters when calves and heifers had repeated episodes of BRD or diarrhea.

## 5. Conclusions

Feeding calves with pasteurized colostrum and milk in the first 21 days of life not only reduces morbidity and mortality, but also reduces morbidity throughout the whole rearing process, increases body weight at first parturition and increases milk yield during the first lactation. Future research should investigate whether a stronger effect can be observed if the number of days of feeding pasteurized cow milk is increased, reducing morbidity and mortality even further and improving body weight and milk production.

## Figures and Tables

**Table 1:** Average (±SEM) and confidence intervals in parentheses (95%) for reproduction, production and health parameters of heifers and cows from the groups fed non-pasteurized (NP) and pasteurized (P) colostrum and milk during the first 21 days of life.

	Treatment Group		
Parameter	NP Group (*n* = 134)	P Group (*n* = 127)	*p*-Value
*Animals*			
Heifer (*n*)	134	127	NA
1st lactation cows (*n*)	110	106	NA
2nd lactation cows (*n*)	90	94	NA
3rd lactation cows (*n*)	71	68	NA
*Reproduction*			
Average AI for 1st pregnancy (*n*)	1.54 ± 0.08 (1.39–1.70)	1.55 ± 0.08 (1.40–1.71)	0.91
Average AI for 2ndpregnancy (*n*)	2.35 ± 0.21 (1.95–2.75)	2.46 ± 0.20 (2.04–2.88)	0.73
Average AI for 3rd pregnancy (*n*)	2.54 ± 0.23 (2.08–3.01)	2.32 ± 0.19 (1.93–2.71)	0.63
Age at 1st parturition (d)	741.4 ± 5.1 (730–745)	737.6 ± 3.7 (731–751)	0.99
Int. calving-calving 1st–2nd Lact. (d)	398.8 ± 7.7 (383.5–414.0)	401.0 ± 5.4 (390.1–411.8)	0.98
Int. calving-calving 2nd–3rd Lact. (d)	415.3 ± 7.4 (400.5–430.1)	399.0 ± 5.5 (388.1–410.0)	0.19
*Production*			
BW at birth (kg)	38.7 ± 1.1 (38.6–38.9)	38.6 ± 1.1 (38.8–38.4)	0.98
BW at 1st parturition (kg)	492.6 ± 3.8 ^a^ (485–500)	531.7 ± 3.8 ^b^ (524–539)	<0.01
BW at 2nd parturition (kg)	575.1 ± 4.6 ^a^ (565–584)	602.0 ± 4.6 ^b^ (593–611)	<0.01
BW at 3rd parturition (kg)	626.4 ± 5.3 ^a^ (615.7–637.0)	645.9 ± 5.3 ^b^ (636.9–654.9)	<0.01
1st lactation 305-d milk yield (kg)	9574 ± 209 ^a^ (9158–9989)	10,147 ± 161 ^b^ (9828–10,461)	0.04
2nd lactation 305-d milk yield (kg)	11,193 ± 314 (10,569–11,817)	11,754 ± 168 (11,419–12,088)	0.66
3rd lactation 305-d milk yield (kg)	12,417 ± 221 (11,976–12,858)	12,515 ± 122 (12,271–12,759)	0.68
*Health*			
1st lactation average of SCC (SCC/mL)	107 ± 20 (67–149)	142 ± 22 (97–187)	0.11
2nd lactation average of SCC (SCC/mL)	120 ± 25 (70.5–169)	162 ± 36 (90–235)	0.22
3rd lactation average of SCC (SCC/mL)	174.5 ± 25.3 (124.0–225.0)	195.4 ± 26.6 (142.1–248.8)	0.27
*Heifers that did not reach 1st parturition*			
BRD morbidity 1st year of life (*n*, %)	13 (56.5)	8 (38.1)	0.28
Diarrhea until 180 days of life (*n*, %)	6 (26.1) ^a^	1 (4.8) ^b^	0.04
*Heifers that reach 1st parturition*			
BRD morbidity 1st year of life (*n*, %)	24 (21.8) ^a^	11 (10.4) ^b^	0.02
Diarrhea until 180 days of life (*n*, %)	14 (12.7) ^a^	5 (4.7) ^b^	0.03
*Culling*			
Heifers that did not reach 1st parturition *			
Slaughtered (*n*, %)	13 (56.5)	17(81.0)	0.61
Dead on the farm (*n*, %)	10 (43.5)	4 (19.0)	0.08
Average days of life at culling (d)	489.4 ± 78.5	653.1 ± 60.1	0.07
*Heifers that reached 1st parturition*			
Slaughtered (*n*, %)	(326.6–652.2)	(526.7–781.1)	0.85
Dead on the farm (*n*, %)	37 (88.1) 5 (11.9)	17 (77.3) 5 (22.7)	0.26
Average days of life at culling (d)	1265.2 ± 51.4 (1161.2–1369.1)	1234.1 ± 47.8 (1134.8–1333.4)	0.62

^a, b^ Values within a row with different superscript letters are significantly different (*p* < 0.05). * There is no information about one heifer in Group NP that did not achieve first parturition. Avg, average; Int, interval; BW, body weight; SCC, somatic cell count; BRD, bovine respiratory disease; NA, not-applicable.

## Supplementary Materials

The following are available online at https://www.mdpi.com/2076-2615/10/9/1494/s1, Raw data of this study are available in Supplementary S1 for interested readers.
