# Supplementary material for: Feeding Calves with Pasteurized Colostrum and Milk Has a Positive Long-Term Effect on Their Productive Performance
Source: Animals (Basel). 2020 Aug 24;10(9):1494. doi: 10.3390/ani10091494 (PMC7552247; doi:10.3390/ani10091494)
Supplement: Supplementary file 1 [file animals-10-01494-s001.pdf]

PART Y/N	AI0+	PART1	AGE1	BW1	MY 1	SCC1	AI1+	BW2	MY2	SCC2	INT 1-2	AI2+	BW3	MY3	SCC3	INT2-3	CULLREAS	STP	PAST	N BRD	N DIAR	DEST	LIFED	DIML	MYLIFE
0		NO															1	7,4	0	1		0	429		
1	1	42766	729	528	8539,0	30	2	627	8928,2	30	361	1	638	13328,7	143	362		8,2	0						
1	2	42771	791	550	9534,6	21	4	606	9953,9	33	550							7	0						
1	1	42766	759	539	12202,0	24	1	610	12278,7	93	367	1	652	11607,7	201	407		7,6	0						
0		NO															1	7,2	0			0	458		
0		NO															1	6,8	0	1		0	1031		
1	3	42666	781	597	10801,3	126	2	682	10677,8	463	383	8	707	10370,0	72	554		7	0					780	27416
1	2	42851	785	541	11100,2	54	1	539	12479,7	21	355	3	576	13115,0	302	402		8	0					624	24260
0		NO															1	6,6	0			0	390		
1	2	42850	797	478	10670,4	53	2	530	12664,0	1082	445							7,6	0					582	21590
1	3	42657	774	581	9765,6	118	4	657	11437,1	153	439	4	693	12810,0	120	440		6,6	0					879	31353
1	1	42636	689	555	12516,9	102	1	618	13337,2	60	362	4	644	12883,6	172	427		7,6	0					737	31316
1	1	42716	683	463	7848,2	24	3	528	9362,8	178	448	1	648	13115,0	98	369		8,4	0					799	23710
1	1	42599	735	437	8628,9	39	1	563	9497,8	100	344							6	0					801	24103
1	1	42859	753	548	9048,0	62	2	570	10716,2	419	373	2	623	12505,0	83	376		8	0	1				633	20705
1	2	42690	726	537	10033,4	26	1	625	12703,4	30	357	1	630	12689,7	90	365		7,4	0	1				689	25989
1	1	42759	721	520	8706,0	341	1	592	11817,5	72	369	1	617	11306,1	37	368		8	0					757	25889
0		NO															6	7,2	0	2		1	460		
1	1	42711	714	493	8681,4	182	2	544	12011,9	46	389	2	647	11895,0	201	390		7,4	0					792	27536
1	2	42654	725	512	8897,2	34	1	610	11537,7	56	367	2	653	11895,0	51	360		7,6	0					1045	35922
1	1	42731	724	493	10963,3	60	3	599	13294,5	19	371	5	597	12810,0	101	438		7	0					537	20901
1	1	42644	737	482	10582,1	46	5	613	11946,0	32	457	5	645	13115,0	123	453		6,8	0	1				853	31879
0		NO															1	6,8	0			0	898		
1	1	42763	698	445	7063,8	21											2	7,8	0			0	1129	431	9982
1	3	42806	804	536	12597,2	22	5	535	12277,8	55	477							7	0					617	25274
1	1	42677	751	545	10605,1	67	1										1	8	0	1		0	1121	301	10466
1	1	42765	757	507	10376,2	28	6	587	10368,1	175	402	4	638	12810,0	72	437		8,2	0					712	24613
1	3	42715	762	523	12296,0	91	12	565	12590,7	105	355	5	621	12200,0	59	453		8	0					841	34265
1	2	42954	810	625	11305,6	317	1	683	12930,4		377							7,2	0					463	17972
1	1	42764	693	485	13450,3	119	4	507	13802,8	349	370	5	580	13646,8	171	453		8	0	1				700	31298
1	1	42762	740	513	11899,0	297	9	570	13796,9		598							8	0					661	26553
1	1	42715	702	535	10373,9	26	1	591	11444,9	27	367	1	625	13289,3	X	366		7,8	0					655	23698
0		NO															2	8,4	0			0	982		
0		NO															1	7,2	0			0	902		
1	4	42656	778	534	10526,5	21	1	620	12672,1	16	360						5	7	0			1	1503	673	25795
1	2	42873	773	493	11128,9	43	4	547	11684,6		481							7,6	0					555	20486
1	1	42599	745	489	10086,3	129	3	605	13330,8	16	386	1	591	11414,6	450	367		6,9	0					751	28560
1	1	42633	702	541	10365,4	38	2	642	9860,1	52	382	4	673	13255,3	301	561		7	0					838	28196
1	1	42626	715	539	8587,2	1353	1	654	7736,2	801	363						2	6,6	0			0	1514	746	19787
1	1	42765	785	546	11021,6	54	3	596	12512,9	929	438	3	647	12654,7	207	401		7,2	0					705	27086
1	1	42678	725	546	3435,0	47											2	7,2	0			0	908	183	2061
1	1	42654	738	524	8760,4	19	1	573	11247,4	17	367	1	648	12196,6	106	360		7	0	1				701	23606
1	1	42731	718	523	10103,7	54											1	7,2	0			0	1246	528	17491
1	1	42667	737	491	9394,1	1266											1	8	0			0	1208	471	14507

0		NO															1	7	0			0	400		
1	1	42768	799	545	10864,1	67	3	576	13431,2	231	405	1	624	13141,2	1132	360		7,4	0					750	29926
1	1	42751	695	468	8579,7	521	4	585	10983,4	26	449	1	632	12810,0	98	369		7,4	0					721	23216
1	2	42799	756	496	9324,4	24	5	539	11221,9	59	435	2	587	12751,0	106	395		8	0					651	21725
1	1	42592	697	447	7721,2	131	4	631	9420,1	190	444	4	668	12900,5	937	462		7	0					932	28057
1	2	42693	726	556	12036,3	15	2	665	13186,3	59	394	1	680	12505,0	615	325		7,6	0					940	38876
1	2	42712	734	542	11481,1	59	1	656	12398,7	98	367	4	687	11984,6	67	431		6,8	0					844	33189
1	1	42805	709	451	9623,4	66	1	541	10859,8	206	355	3	587	12822,2	40	416		7	0					693	23917
1	2	42595	619	505	10424,2	24	2	576	10182,1	21	358							7,2	0					769	25958
0		NO															1		0			0	190		
1	1	42622	736	527	8561,3	51											1	6,8	0	2		0	1539	803	22540
1	1	42625	695	567	12093,1	32	8	656	15788,9	500	507						3	6,6	0			0	1305	488	20597
1	4	42629	721	552	9268,1	231	2	578	10654,3	13	361						3	6,6	0			0	1344	446	14089
1	1	42758	738	557	12956,7	51	1	587	11090,2	292	375						1	7,8	0			0	1448	599	23396
1	1	42820	723	469	10855,6	111	2	531	12048,6	253	380	1	577	11662,6	127	387		7,6	0		1			665	24915
1	1	42842	754	512	8150,6	84	6	629	11337,9	52	484							8	0		1			573	16880
1	1	42850	752	472	11247,3	22	2	567	12931,0	357	518							8	0					561	21996
1	1	42758	696	531	12470,5	20	1	551	12393,3	33	374	5	593	11426,4	203	446		7,8	0					731	29565
1	2	42731	672	585	11078,6	194	2	618	13111,4	35	396	2	661	14662,2	56	403		7,4	0					768	31297
1	1	42702	749	539	10683,8	25	1	600	12974,8	32	364	1	654	14663,5	204	371		6,8	0					659	25914
1	1	42681	731	552	9251,4	64	1	622	13530,9	32	402	1	658	13138,5	X	367		6,8	0					680	25279
0		NO															1	7,2	0			0	772		
1	1	42629	742	507	10190,2	177	1	562	13147,9	86	361	2	598	12238,1	180	390		6	0	1				727	28076
1	1	42581	707	489	5037,1	98	1	610	10721,4	41	375	5	663	12572,8	145	475		7	0					758	21346
0		NO															11	6,4	0		1	1	179		
1	1	42859	719	544	9633,6	25											2	7,6	0			0	970	251	7928
1	1	42793	707	529	7820,0	451	4	625	9964,3	120	468	1	654	11895,0	39	379		8	0					722	21239
1	2	42629	743	594	5276,8	523											2	7,4	0	1		0	732	628	10865
1	1	42886	747	540	10629,7	25	2	606	9837,0	23	388	1	634		X	382		8	0						
1	1	42660	717	530	6640,4	43	1	630	8959,5	20	360	1	655	9483,0	145	369		6,6	0					699	18318
1	2	42603	737	525	9635,3	113	1	634	10371,7	735	390	3	671		X	413	10	6,8	0			0	1566		
1	1	42566	689	610	11386,7	175	1	643	12076,4	171	358	3	711	12993,0	207	412		6,8	0					788	30943
1	1	42661	716	524	10188,2	25	1	567	10816,3	68	369	2	685	12092,4	110	395		8	0					694	24172
1	1	42270	700	491	9637,8	226	1	586	12107,6	56	359	1	604	12702,9	351	365		7,2	0					908	34183
1	2	42292	726	511	12510,6	28	2	584	14975,7	17	424	1	616	13543,6	139	371		7,2	0					1007	44929
1	3	42844	812	560	12529,7	43	4	627	14034,5	120	430							6,8	0					573	24536
1	1	42792	697	530	11843,0	19	1	580	12035,1	30	364	3	616	12291,5	337	411		6,6	0	1				715	28097
1	1	42633	691	495	7694,7	117											5		0			1	922	210	5298
1	2	42807	730	539	8702,0	76											1	7,6	0			0	1348	708	20200
0		NO															1	7,8	0			0	508		
1	1	42765	726	543	9491,3	95	3	592	12419,2	34	409	3	648	12020,4	407	391		7,8	0					762	27499
1	1	42609	730	497	10829,9	67	1	588	16136,5	95	366						5	7,4	0			1	1278	495	20743
1	3	42828	776	446	9148,1	34	1	510	10333,8	50	370	3	707	11709,6	529	362		7,8	0					693	22723
0		NO															1	7,6	0			0	865		
0		NO															1	8	0	1		0	403		

1	2	42922	797	606	10828,7	151	5	639	10872,8	2765	443							8	0					496	17626
1	1	42571	715	530	11974,8	28	1	567	13488,9	21	351	1	594	11182,8	140	363			0					794	32172
1	1	42853	717	517	11449,3	28	3	598			410						5	7,6	0		1	1	1139		
1	3	42828	785	553	10245,7	156	6	634	13428,7	79	508							8,2	0					584	21079
1	2	42620	761	580	11682,8	49	1	620	11348,4	66	360	4	693	13754,0	173	458		8,2	0					755	28762
1	3	42697	816	583	6710,8	125						3	708	12505,0	125	412		8	0					1083	29566
1	1	42757	711	542	6728,3	64											1	7,8	0			0	1394	683	15067
1	3	42690	815	565	11333,4	39	1	617	12691,5	54	363	2	690	12390,6	X	403		6,6	0		1			678	26813
1	3	42697	821	534	11358,0	142	1	579	13330,9	54	364	1	590	13496,3	90	363		6,8	0					673	27447
1	3	42686	769	500	9905,5	107	1	603	10420,2	229	360	1	627	10691,9	23	362		7,2	0					688	23053
1	3	42863	740	518	11218,7	129	6	617	10998,1	80	482								0					558	20427
0		NO															6		0	3		1	735		
1	1	42629	737	586	9659,6	495	2	624	12171,8	104	381	2	659	12805,6	182	400		7,4	0					744	27135
1	2	42857	770	571	6810,7	47	1	686	6865,7	49	367						2	7,8	0			0	1184	350	7824
0		NO															1	6,2	0	2		0	907		
0		NO															6	6,8	0	3		1	450		
1	2	42656	718	586	11379,5	17	3	641	10496,6	18	429	1	673	9500,2	X	368		7,4	0					701	25102
1	1	42807	743	508	10861,4	24	1	590	11450,5	30	367	1	653	11895,0	94	361		7,8	0					720	26553
1	1	42662	707	575	12061,8	73	1	648	12555,5	68	357	1	652	13426,1	172	361		7	0					711	29054
1	1	42675	691	592	10826,5	521	1	695	12892,4	44	360	1	651	15200,9	98	358		7,6	0					697	28021
1	2	42759	727	563	11080,0	826	1	604	10212,6	584	364	1	681	12810,0	402	366		7,8	0					805	29406
1	4	42751	808	543	9801,3	120	2	603	9633,2	56	376	4	668	12166,5	72	445		7	0					779	25543
1	1	42729	702	566	12000,0	81	1	630	13110,0	45	356	1	648	11643,8	X	364		6,8	0					644	26408
1	1	42726	692	527	11167,3	259	1	600	11802,4	31	375	2	657	12351,6	208	397		9	0					802	30684
1	1	42693	697	573	11028,3	1148	2	678	13551,7	88	401	6	714	11854,0	74	510		7	0					797	32218
1	3	42808	793	586	10963,6	44	6	674	12329,3	37	524							7,8	0					615	22783
1	2	42797	817	505	9665,5	204											6	7,2	0	1		1	1059	242	7669
1	1	42761	681	548	11135,5	72	1	605	12781,1	56	448	2	653	12437,2	X	412		7,4	0		1			603	23609
1	1	42822	723	484	9918,7	43	2	536	10793,8	27	376	1	593	13115,0	61	368		8	0					701	24631
1	1	42810	707	484	10927,9	215	6	478	6851,4	14	477						8	7,8	0			0	1294	537	17770
0		NO															1	7	0	1		0	919		
0		NO															1	7,8	0			0	1011		
1	1	42811	714	587	8608,8	24	5	670	12015,0	26	522							7,4	0					611	18921
1	2	42838	754	601	6653,4	88	2	707	8579,1	106	380	1	729	13493,6	89	358		6,8	0					632	17411
1	2	42878	775	578	11803,9	89	2	634	13024,2	84	400	1	682		X	369		7,2	0						
1	1	42910	779	542	12388,7	XX	1	584	13779,4	56	360	2	647		X	381		7,8	0						
1	1	42886	735	553	9995,8	23	3	585	11485,6	29	413							7,4	0					528	18203
0		NO															1	7,4	0			0	844	0	0
0		NO															1	7,9	1	1		0	548	0	0
0		NO															1	7,4	1			0	795	0	0
1	3	42403	757	519	10852,9	44	1	604	13104,2	54	357	1	656	13281,4	98	373	3	7,8	1			0	1597	708	28357
0		NO															8	6,6	1	1		0	240		
1	1	42390	779	468	11231,0	32	3	615	12084,4	47	392	8	637	14898,3	104	577			1					991	39429
1	1	42288	731	520	6345,1	26											1	7	1	2		0	1245	514	10693
1	2	42407	712	475	10582,5	46	2	590	12072,4	73	385	2	655	10886,5	143	417		9	1	3				968	35623

1	2	42482	782	480	10302,1	602	10	529	10645,1	597	563						3	7,6	1			0	1396	554	18770
1	1	42444	744	457	11925,5	93	1	523	703,1	84	358						2	7	1	2		0	1161	369	12257
1	1	42291	699	496	10281,6	19	1	563	13198,0	415	375	3	582	13431,0	109	358		7,2	1					965	39164
1	1	42759	1118	414	8877,5	19	1	486	9791,9	18	2						3	7,6	1			0	1187	386	11436
0		NO															6	7,4	1	3	1	1	7		
1	1	42450	740	477	10290,4	33	1	562	11479,4	173	371	6	610	14837,4	204	492		7	1					921	35296
1	1	42428	703	476	9492,7	293	3	568	12441,0	66	388	1	600	12603,3	187	333	3	7,2	1			0	1497	948	35541
1	1	42400	723	521	11700,0	306	1	610	13502,9	79	366	2	683	14112,5	134	388		7	1					979	42114
1	1	42457	733	549	9809,3	36	6	647	11315,8	67	517	5	679	13215,3	110	436		7,6	1					927	32585
1	1	42379	721	450	7241,1	43	2	612	5097,4	48	392						8		1			0	1243	441	9760
1	2	42412	762	440	9436,9	90	1	496	10891,2	125	373	10	580	11070,9	108	557		7,2	1		1			929	31726
1	2	42388	699	470	10813,3	19	3	570	11150,0	45	407	5	680	13416,5	78	602		8,2	1	1				992	36500
1	2	42562	746	486	8722,5	156	7	620	11653,0	146	512	1	634	12498,1	43	365		6,2	1					812	26745
0		NO															1	6,4	1	1		0	745		
1	1	42326	770	498	9462,6	42	5	548	11557,6	33	456	1	620	11437,5	387	359		7,4	1					1053	37020
1	1	42348	812	494	9531,7	32	1	514	9752,5	20	515	1	657	6641,6	74	386	2	6,4	1			0	1511	730	22529
1	1	42418	711	389	9547,9	33	7	520	12733,0	42	538							9,2	1		1			1003	36902
1	1	42610	794	549	11145,6	105	1	568	11310,0	169	337	1	620	12705,3	207	370		7,6	1					762	28859
0		NO															1	7,4	1	2	1	0	960		
0		NO															9	7,4	1		1	1	211		
1	2	42523	851	480	8350,4	139											12	8	1	1		0	888	37	1013
0		NO															6	7	1	1	1	1	216	0	0
1	1	42284	722	570	2802,2												3	7,4	1	1		0	770	48	441
1	2	42562	727	480	6638,3	34											2	7,4	1			0	1450	723	15736
1	2	42628	793	586	14325,1	24	1	620	14270,2	19	364						4	7,4	1			0	1303	454	21297
0		NO															6		1	2		1	15		
1	1	42379	716	536	11289,5	16	2	608	13658,0	22	392	1	690	12115,3	56	373		7,6	1		1			962	38835
1	2	42390	675	419	8676,3	121	1	576	488,0		419						5	7,4	1	1		1	1089	316	8855
1	1	42379	710	498	10790,0	140	3	615	10828,5	57	392							8,2	1					603	21370
1	1	42404	727	462	9498,2	24	NO				365						6	6,8	1	2		0	1175	480	14948
1	1	42362	722	485	8136,7	53	3	573			358						2	7,8	1			0	1631	931	24837
1	3	42873	746	512	10300,4	72	5	587			481						2		1	1		0	1264	311	10503
1	2	42595	744	517	10887,7	22	1	544	12759,6	86	378	2	604	13507,8	107	378		7	1					786	31199
0		NO															1	7,6	1	1		0	1405		
1	3	42338	785	537	8155,8	206											2	7,2	1	1		0	1732	947	25323
0		NO															1	7,2	1			0	833		
1	1	42361	808	524	10730,5	35	2	561	10244,1	44	374	3	621	12988,4	78	524		7,8	1					1007	36364
1	2	42348	795	532	8628,6	42	6	585	11825,2	35	515	2	640	12713,0	109	400		7,4	1					972	33924
1	1	42383	706	454	9356,0	223	3	540	12187,2	395	591	2	535	12494,3	162	395		6,8	1					986	34510
1	4	42379	799	478	6694,9	57											4	8	1			0	880	81	1778
1	1	42362	716	483	9133,5	21	1	583	8553,0	37	358							7,6	1	1				1067	30521
1	1	42285	717	472	11566,9	37	2	486	5987,9	218	393						2	6,4	1	2	1	0	1717	964	25456
1	1	42275	702	519	9380,7	43	3	570	12356,4	256	346	1	654	12428,8	307	421		8,4	1					963	35641
1	1	42386	764	462	9465,5	79	1	573	10807,3	202	360	1	618	10026,1	1145	365		7,6	1					989	32716
1	1	42349	761	571	7844,8	1940											4	7	1	1		0	872	111	2855

1	1	42467	739	476	9738,2	133											1	6,4	1			0	1424	685	21871
1	1	42466	694	503	9478,8	16	1	562	11415,1	17	340	2	693	13483,4	109	435		8	1					894	33153
1	2	42377	811	493	4187,4												3	6,6	1	1		1	859	48	659
0		NO															1	xx	1	2		0	819		
1	1	42550	722	485	9937,2	180	3	566	11999,5	13	421						3		1			0	1394	616	21767
1	1	42438	774	419	3167,7	58											1	6,6	1			0	831	57	592
1	1	42393	714	530	11887,7	45	1	609	14062,6	66	381	1	630	13020,0	181	359		7,4	1					963	40929
1	1	42333	762	504	10157,5	109	1	570	12192,5	127	374	2	590	11255,9	134	379	1	8,4	1			0	1840	975	35823
1	1	42436	766	422	2961,0	132											8	7,4	1	1		0	790	24	233
0		NO																7	1						
1	1	42303	695	453	7138,1	37	1	574	10725,3	65	387	1	649	10597,4	201	373	1	8	1			0	1860	1046	32708
1	1	42219	657	521	6906,0	19	1	599	8150,0	72	379	3	657	8751,6	132	436		7	1					1007	26398
1	1	42265	692	433	9508,7	49	2	539	12485,2	40	384	1	615	13426,1	67	366		7,6	1					942	36214
1	2	42352	781	431	2099,1												2	6,4	1	1		0	805	34	234
1	1	42265	688	523	9575,4	23	7	621	12672,8	32	502	7	723	15394,0	167	525			1					1041	40178
1	1	42273	697	496	10126,3	112	4	527	11445,9	45	435						3		1			0	1646	730	25738
1	3	42404	732	489	12771,3	29	1	534	15076,1	22	365	1	585	14795,2	34	363		6,8	1					962	44858
1	3	42359	778	503	11563,6	70	4	564	14381,9	26	501	4	599	14952,1	102	439		6,6	1	1				1008	43687
0		NO															6	7	1	2		1	319		
0		NO															6		1	1			15		
0		NO															7		1		1		10		
1	2	42624	797	558	11690,0	90	1	628	14128,7	54	360	2	659	12932,5	64	371		7,4	1					731	30965
1	2	42587	766	490	11541,3	13	1	545	15038,6	1066	362						3	7	1			0	1317	496	20936
1	4	42389	810	478	0,0												10	6,2	1			1	813	3	
1	2	42366	744	488	9582,6	469	6	587	8552,5	1218	478							8	1	1				1055	31012
1	3	42342	770	463	9585,1	26	1	539	11112,6	22	358	1	582	11803,4	304	365		7	1					1016	36393
1	1	42261	727	497	8020,4	198											4	7,4	1			0	926	199	5233
1	2	42316	707	488	8970,1	14	1	534	11677,3	11	330	1	547	12079,6	39	392		8	1					927	33123
1	1	42383	713	492	11182,5	67	3	557	13850,0	42	409	1	577	12805,1	139	370		6,6	1					976	40122
1	1	42689	722	583	10001,3	36	3	670	11106,7	30	411						2	6,6	1			0	1430	655	22551
1	1	42614	783	560	9788,2	294	3	658	12088,1	119	415	2	670	12205,0	100	386		7	1					745	26858
1	2	42331	736	506	9735,6	19	1	587	11417,6	20	381	2	613	11608,9	85	387		8	1					1009	36202
1	1	42443	714	466	10533,8	142	1	524	13394,5	79	357	1	630	12264,0	98	484		7,6	1	2				727	36663
1	2	42420	735	463	11993,9	220	1	581	11911,4	173	378	4	620	15133,4	656	508		7,6	1		1			943	38712
1	3	42449	764	431	8863,5	168	6	526	10840,2	45	502	1	565	13061,4	40	360		7,8	1					911	30696
1	1	42568	770	476	10406,8	51	1	574	12126,3	22	350	1	598	12274,7	121	358		7,4	1		1			796	30065
1	1	42390	758	486	2135,0												11	7,4	1			0	757	23	161
1	1	42502	677	430	10145,4	79	3	529	11591,0	30	346						3	7,4	1			0	1381	649	23029
1	1	42390	699	413	10877,5	61	2	474	7826,2	21	419	4	540	5513,5	X	449		7,6	1					764	23287
1	1	42378	693	463	10594,6	77	1	558	13325,6	64	385	1	600	13092,7	68	371		7	1					983	39696
1	1	42463	670	553	10719,0	37	1	605	11329,2	46	359	3	623	11388,9	107	425		7	1					886	32345
1	1	42338	751	505	8159,0	48	2	612	10729,6	27	371	5	671	13900,0	205	530		6,8	1					1026	35818
1	4	42640	800	550	11787,1	75	1	604	11246,0	41	375	1						6,6	1					772	29017
0		NO									#¡REF!						6	7	1	2		1	219		
1	2	42495	704	470	9747,9	20	2	527	1161,5	29	384	5	597	12582,4	47	467		7	1		1			866	17428

1	4	42354	828	501	10249,7	32	2	545	11711,1	26	466	4	558	12179,9	78	459		8	1		1			1021	37489
1	2	42494	742	482	8298,7	137	1	589	7986,5	1346	370	2	638	10393,7	56	393		7	1					859	24545
1	1	42430	682	450	9544,5	409	1	527	9667,0	176	366	7	578	1616,5	X	554	10	7,4	1	1		1	1612	796	24838
1	1	42533	718	479	9455,7	430	3	646	12546,4	25	465	1	685	13353,5	200	368		6,8	1	1				822	29950
0		NO															1	7,6	1			0	642		
1	1	42605	763	493	8662,2	22	1	554	10724,7	48	369	2	570	11509,0	406	374			1					729	24056
0		NO															5	7,6	1			1	459		
1	3	42485	707	517	10832,5	191	1	583	12697,8	32	362	5	620	12855,3	94	474		7,6	1	1				856	33858
1	1	42275	714	490	9224,8	17	4	559	10167,2	23	421	4	668	12415,5	87	446		6,6	1					1095	37830
1	3	42313	779	516	9102,5	29											2	7	1		1	0	1532	783	23368
1	1	42645	698	537	7399,8	34	3	655	10713,4	23	429	1	681	10065,0	X	363		6,8	1					697	20619
0		NO															1	6,4	1			0	992		
1	1	42361	709	480	10491,3	293	3	565	13374,1	66	417	1	599	12893,6	109	371	3	8	1			0	1570	757	29563
1	1	42432	710	437	10268,3	22	1	534	11695,8	359	380	2	570	12105,7	432	389		8	1		1			904	33589
1	1	42301	701	464	26063,6	121	8	604	12777,2	32	631	2	623	13237,0	50	379		6,8	1					655	34876
1	2	42307	698	498	11183,1	94	4				478						10	8	1			0	1179		
1	1	42374	741	505	11208,8	15	1	590	13103,1	32	367	1	629	13208,6	221	363		6,6	1					960	39344
1	1	42356	725	529	12337,8	57	3	605	15108,4	43	412	1	635	14035,3	478	363		7,6	1					1015	45771
0		NO															1	7,2	1			0	763		
0		NO															11	7,4	1			0	373		
0		NO															1	8	1	2		0	219		
1	2	42522	727	516	10886,1	20	1	625	14040,1	19	358	1	672	15476,4	56	371		7	1		1			829	36098
1	2	42512	745	471	9645,2	16	2	552	1152,3	16	402	1	617	11712,6	93	363		7,4	1					841	19535
1	1	42493	722	462	9957,6	23	3	554	11997,6	50	411	2	594	12080,7	305	388		7,6	1					869	31879
1	2	42626	829	440	10652,4	20	1	480	10699,2	22	364	3	538	12123,8	47	422		8,4	1		1			740	26210
1	1	42452	717	475	9773,8	168	1	612	12280,6	111	348	4	672	13498,4	108	462		8	1	1				928	35680
1	1	42488	736	506	9869,9	54	1	616	10285,7	131	373	5	667	13464,7	125	458		7	1					877	30773
1	1	42788	703	507	8420,7	26												7,6	1					690	19050
1	2	42548	751	504													2	8	1	3		0	755		
1	1	42502	695	509	10688,5	61	1	581	13083,1	40	346	4	676	14713,5	82	441		7,6	1		1				
1	1	42486	700	482	11216,3	81	1	578	13162,3	62	381	2	614	11117,7	1107	396		6,6	1					934	
1	1	42555	721	572	10043,9	18	2	668	11324,0	15	368	2	701	12200,0	56	387		6,8	1						22560
1	1	42500	740	563	11055,3	106	2	627	11834,5	107	381	5	XX			513	10	7,8	1			1	1637	793	29942
0		NO															1	7	1		1	1	451		
1	1	42482	683	496	10722,5	29	2	598	12901,5	44	385	1	613	12840,5	56	373		x	1		1			861	34079
1	3	42591	767	592	11683,8	58	1	685	12837,4	114	349	2	716	12891,3	124	435		6,8	1	1				768	31224
1	3	42614	775	524	14015,4	33											3	6,8	1			0	1173	398	18289
